# LONG-TERM CARE II EFFECTS OF A NEW VALUE-BASED REIMBURSEMENT SYSTEM ON EXPENDITURES AND CARE QUALITY IN MINNESOTA NURSING HOMES

**DOI:** 10.1093/geroni/igz038.2723

**Published:** 2019-11-08

**Authors:** Zachary Hass, Valerie Cooke, Greg Arling

**Affiliations:** 1 Purdue University, West Lafayette, Indiana, United States; 2 Minnesota Department of Human Services, St. Paul, Minnesota, United States

## Abstract

Although several states have implemented Value Based Reimbursement (VBR) systems in long-term care; little is known of their impact. In 2016, Minnesota passed new VBR legislation to tie payment to quality with increased funding earmarked for nursing and other care-related services. We evaluated the effect of the policy change on care-related expenditures and measures of care quality. Data sources were cost reports and quality measures for the years 2013-2017 from 348 Minnesota nursing homes. Analysis consisted of descriptive tables, time plots, and linear growth curve modeling. We found increased expenditures for nursing and care-related services, particularly nursing assistants, during the first year; while quality metrics did not appear to be impacted by the policy change. Some differentiation was seen across facilities in their response to the policy change based on occupancy rate, rural-urban continuum code, attachment to a hospital, and resident acuity. The lack of an improvement in care quality might be attributable to VBR design as the quality incentive affected very few facilities. Additionally, there is the challenge of improving quality metrics even with additional resources. The legislature is currently considering changes to VBR policies in response to the report from this study.

